# Unmasking alternative splicing inside protein-coding exons defines exitrons and their role in proteome plasticity

**DOI:** 10.1101/gr.186585.114

**Published:** 2015-07

**Authors:** Yamile Marquez, Markus Höpfler, Zahra Ayatollahi, Andrea Barta, Maria Kalyna

**Affiliations:** 1 Max F. Perutz Laboratories, Medical University of Vienna, Vienna A-1030, Austria;; 2 Department of Applied Genetics and Cell Biology, BOKU – University of Natural Resources and Life Sciences, Vienna A-1190, Austria

## Abstract

Alternative splicing (AS) diversifies transcriptomes and proteomes and is widely recognized as a key mechanism for regulating gene expression. Previously, in an analysis of intron retention events in *Arabidopsis*, we found unusual AS events inside annotated protein-coding exons. Here, we also identify such AS events in human and use these two sets to analyse their features, regulation, functional impact, and evolutionary origin. As these events involve introns with features of both introns and protein-coding exons, we name them exitrons (exonic introns). Though exitrons were detected as a subset of retained introns, they are clearly distinguishable, and their splicing results in transcripts with different fates. About half of the 1002 *Arabidopsis* and 923 human exitrons have sizes of multiples of 3 nucleotides (nt). Splicing of these exitrons results in internally deleted proteins and affects protein domains, disordered regions, and various post-translational modification sites, thus broadly impacting protein function. Exitron splicing is regulated across tissues, in response to stress and in carcinogenesis. Intriguingly, annotated intronless genes can be also alternatively spliced via exitron usage. We demonstrate that at least some exitrons originate from ancestral coding exons. Based on our findings, we propose a “splicing memory” hypothesis whereby upon intron loss imprints of former exon borders defined by vestigial splicing regulatory elements could drive the evolution of exitron splicing. Altogether, our studies show that exitron splicing is a conserved strategy for increasing proteome plasticity in plants and animals, complementing the repertoire of AS events.

In the majority of eukaryotic genes, the protein coding information of exons is interrupted by intervening sequences, introns. Differential inclusion of exons and introns or their parts in mature mRNAs, so-called alternative splicing (AS), results in multiple transcript and protein variants with different fates and functions from a single gene. About 95% of human and 60% of *Arabidopsis* genes are alternatively spliced (Pan et al. 2008; Wang et al. 2008; Marquez et al. 2012). The repertoire of AS transcripts produced from a single gene is dynamic and changes in different tissues, during development, and in response to environmental cues (Kalsotra and Cooper 2011; Staiger and Brown 2013). Consequently, AS has emerged as a major mechanism to increase the density of information encoded by eukaryotic genomes. Therefore, understanding AS is of paramount importance as further emphasized by linkage of abnormal AS to numerous human diseases, including cancer (Srebrow and Kornblihtt 2006; Kelemen et al. 2013). Nevertheless, storing, retrieval, and processing of AS relevant information remain incompletely understood.

Intron removal relies mainly on the core splicing signals present in every intron: 5′ and 3′ splice sites and branch point (Wang and Burge 2008). However, in *Arabidopsis* and human, these signals represent only part of the information required to define introns (Lim and Burge 2001). Multiple features such as the presence of intronic and exonic splicing regulatory *cis*-elements, length of introns and exons, their differential guanine-cytosine (GC) content, distinct DNA methylation, histone modifications, and positioning of nucleosomes over introns and exons and at exon/intron boundaries contribute significantly to the recognition of the core splicing signals and can change splice site selection, resulting in AS events (Braunschweig et al. 2013; Reddy et al. 2013). Common types of AS events include intron retention (IR), usage of alternative 5′ and 3′ splice sites (A5SS and A3SS), exon skipping (ES), and mutually exclusive exons, whereby IR is a frequent event both in human and in plants (Ner-Gaon et al. 2004; Marquez et al. 2012; Braunschweig et al. 2013, 2014; Reddy et al. 2013). IR events stall expression of certain genes at particular stages, cell types, or conditions and therefore are thought to control developmental transitions or stress responses (Boothby et al. 2013; Wong et al. 2013; Braunschweig et al. 2014; Shalgi et al. 2014). In spite of their prevalence and functional impact, many questions concerning IRs remain unanswered.

Previously, we conducted a genome-wide survey of the features of retained introns in *Arabidopsis thaliana* (Marquez et al. 2012). This analysis revealed a subfamily of IRs that constitute internal regions of annotated protein-coding exons, which we referred to as cryptic introns (Marquez et al. 2012). These introns possess all the canonical core splicing signals (5′ and 3′ splice sites and branch point) and, as they are internal parts of the protein-coding exons, they do not contain stop codons. On the basis of their exonic and intronic nature, here we name them exitrons (exonic introns [EIs]) and define them as alternatively spliced internal regions of protein-coding exons. As exitrons are protein-coding sequences directly flanked by protein-coding exonic sequences, they have a great potential to boost protein diversity via AS. Furthermore, these intrinsic features of exitrons raise questions about the origin and evolution of their splicing. Here, we present a comprehensive characterization of this AS event in *Arabidopsis* and human.

## Results

### Exitron splicing, an alternative splicing event inside protein-coding exons

**Figure 1.**
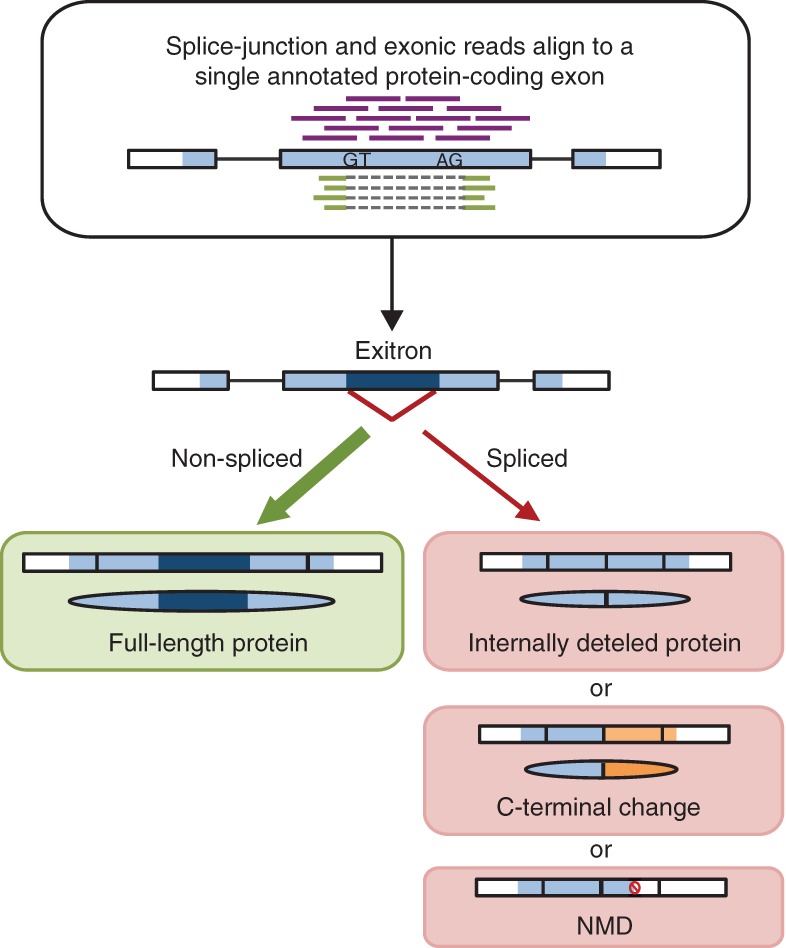
Identification of exitrons (EIs) and consequences of their splicing. Splice junction and exonic reads aligning to a single annotated protein-coding exon were used to identify EIs. As an EI (dark blue) is an internal part of a protein-coding exon, a full-length protein is produced when the EI is not spliced out (shown by a thicker green arrow, as EI-containing transcripts are the major isoforms). Splicing of an EI with a length of a multiple of three results in an internally deleted protein isoform. Splicing of other EIs leads to a frame-shift downstream from the splice junction and results in changed protein C-termini (orange) or can produce NMD-sensitive transcripts (red No sign indicates premature termination codon [PTC]).

**Figure 2.**
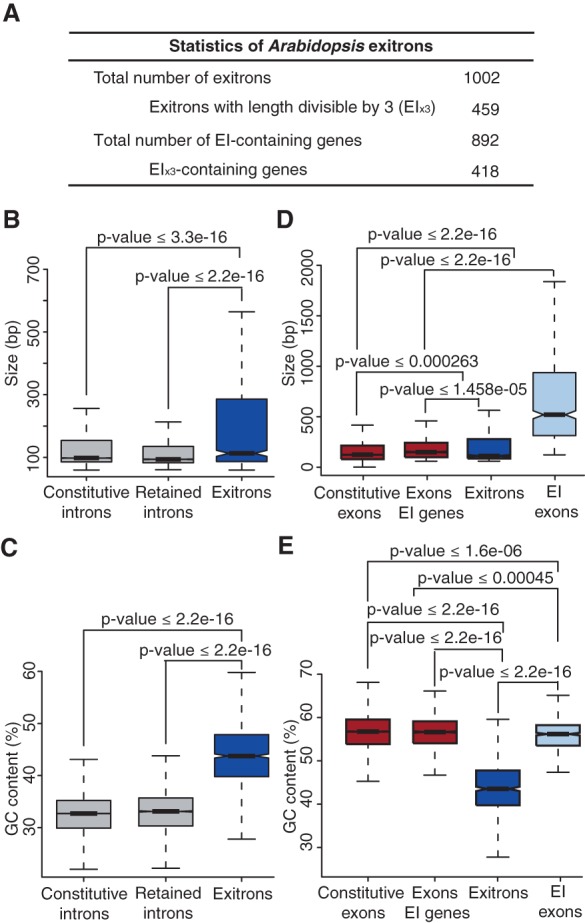
Statistics and features of *Arabidopsis* EIs. (*A*) General statistics of *Arabidopsis* exitrons. (*B*,*C*) Comparisons of size distribution (*B*) and GC content (*C*) of exitrons and introns. (*D*,*E*) Comparisons of size distribution (*D*) and GC content (*E*) of exitrons and exons. (*B–E*) Data presented as Tukey box plots.

Overlapping splice junction and exonic reads (Fig. 1) derived from our *A. thaliana* RNA-seq (flowers and 10-d-old seedlings) (Marquez et al. 2012) mapping to a single annotated protein-coding exon were used to identify EIs and to distinguish them from other IRs (Supplemental Methods). We have defined a set of 1002 exitrons in 892 *Arabidopsis* genes (Fig. 2A; Supplemental Table 1). As expected from our previous analysis (Marquez et al. 2012), *Arabidopsis* exitrons have weaker splice site signals than other introns (Supplemental Fig. 1). Intriguingly, 18.9% of exitrons are located in 165 genes annotated as intronless (Supplemental Table 1), suggesting exitron splicing (EIS) to be a novel source of alternative transcripts and protein isoforms for these genes. In total, EIS affects 3.3% of *Arabidopsis* protein-coding genes (27,206; TAIR10). The exitron subset constitutes 11% of all IRs (9142) and 3.7% of all AS events detected in the same sample (Marquez et al. 2012). We validated EIS events, including those in annotated intronless genes, by various methods (Supplemental Results; Supplemental Figs. 2–4, 6; Supplemental Tables 2–4).

As exitrons constitute intraexonic regions and were identified in the pool of IRs, we next asked how similar are they to exons and introns and, importantly, to IRs. *Arabidopsis* exitrons are overall longer and have a higher GC content than IRs and constitutive introns (Fig. 2B,C). However, their sizes are closer to the sizes of constitutive exons and other exons of the EI-containing genes (Fig. 2D). Interestingly, EI-containing exons tend to be longer than other exons (Fig. 2D). The GC content of exitrons is lower than in all groups of exons (Fig. 2E), indicating also that this is a specific feature of exitrons and not a general property of EI-containing exons or genes. This lower GC content in exitrons reflects their intronic nature and may be important for EIS. These results show that exitrons possess properties differentiating them from constitutive exons and introns and from IRs.

Furthermore, in contrast to IRs, where a premature termination codon (PTC) is very often generated in the retained intron sequence or in the downstream exon, nonsplicing of exitrons never introduces any stop codons as they are protein-coding sequences. It is only upon exitron splicing that changes in the fate of the resultant transcripts can occur. Splicing of exitrons with lengths of multiples of 3 nucleotides (nt) (EI_x3_) leads to the removal of an internal protein sequence (Fig. 1). Splicing of non-EI_x3_ exitrons changes the reading frame, which either alters protein C termini or introduces a PTC downstream from the splice junction, potentially triggering nonsense-mediated RNA decay (NMD) (Fig. 1). Indeed, we found that PTC+ EI-spliced transcripts were elevated in the NMD mutants (*upf1-5* and *upf3-1*) that accumulate NMD-sensitive transcripts. In contrast, no changes in the abundance of EI_x3_-spliced transcripts were detected (Supplemental Results; Supplemental Fig. 4). Therefore, EIS may both affect protein abundance by targeting transcripts to NMD or increase protein diversity via EI_x3_ splicing.

Notably, by analyzing our RNA-seq data set for seedlings and flowers (Marquez et al. 2012), we find that the fraction (45.8%) of the *Arabidopsis* EI_x3_s (Fig. 2A) differs significantly from the expected frequency of one-third in the absence of selective pressure and from the 31.7% and 33.1% 3n (length of multiples of 3 nt) fractions of IRs and constitutive introns, respectively. Moreover, it has been shown that the frequencies of the 3n introns without stop codons are low in all tested eukaryotes, implying a strong negative selection (Jaillon et al. 2008). In contrast, the EI_x3_ fraction (45.8%) is close to the 46.3% 3n fraction of exons that we identify in *Arabidopsis*. In general, 3n exons are preferred from plants to primates (43%–47%) (Tomita et al. 1996). This indicates that exitrons and exons are under a similar evolutionary pressure to preserve the reading frame.

### Exitron splicing in *Arabidopsis* is regulated in tissues and by stress

A gene ontology (GO) classification of EI_x3_-containing genes (the set where exitron splicing certainly results in protein isoforms as it does not introduce PTCs) revealed an enrichment of genes involved in stress response, transcription and developmental processes, and nucleotide and protein binding or kinase activity (Supplemental Fig. 5), implying a regulatory role for EIS in plant adaptation and development.

**Figure 3.**
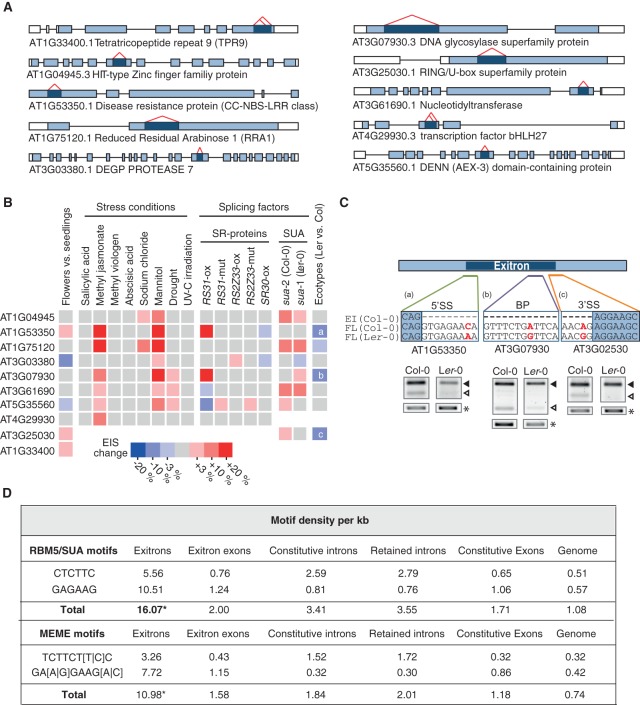
Regulation of exitron splicing in *Arabidopsis*. (*A*) Structures of EI_x3_-containing genes tested by RT-PCR in *B*. Dark blue indicates exitron; red carets, exitron splicing. (*B*) Heatmap for EIS in different tissues, stress conditions, splicing factor mutant and overexpression lines, and Col-0 and Ler-0 ecotypes. The coloring represents only significant changes (*P*-value ≤ 0.1). a, b, and c refer to cases described in *C*. (*C*) SNPs affect EIS in Ler-0 ecotype. SNPs in the splicing signals are indicated in red. 5′SS, 3′SS, and BP indicate 5′ splice site, 3′ splice site, and branch point, respectively. RT-PCR products of the full-length (FL) and EI-spliced isoforms (filled and open triangles, respectively) are shown. Ubiquitin was used as a loading control (*). (*D*) RBM5/SUA motifs and MEME-predicted motifs are enriched in exitrons ([*] *P*-value < 0.0001).

To assess whether EIS can be regulated during development and in response to stress, we tested 10 randomly chosen EI_x3_-containing genes (Fig. 3A) by RT-PCR. We found that the ratios of the full-length and EI-spliced isoforms differ in seedlings and flowers (Supplemental Results; Fig. 3B; Supplemental Fig. 6A,B). Moreover, the proportion of some EI-spliced isoforms reaches up to 50% of the total transcripts, indicating that EI-spliced and EI-containing transcripts can be abundant under normal growth conditions. These results suggest a role for EIS in the regulation of protein diversity in plant tissues at different developmental stages. We next examined EIS under a variety of stresses (Supplemental Results; Fig. 3B; Supplemental Fig. 6C–E). For each gene tested, EIS was significantly affected in at least one condition, with methyl jasmonate (involved in plant defense and development) and mannitol (osmotic stress and plant defense) showing the broadest effect. These differential changes suggest that EIS regulation is not the result of a general stress response but rather is specific to certain stresses, thus further supporting a role in plant adaptation.

### Exitron splicing responds to changes in levels of splicing factors in *Arabidopsis*

We next investigated whether EIS can be affected by splicing factors. Serine/arginine-rich (SR) proteins influence a variety of AS events in plants (Reddy et al. 2013). We analyzed EIS of the same set of EI_x3_-containing genes in different *Arabidopsis* SR protein mutant and overexpression lines (Supplemental Results; Fig. 3A,B; Supplemental Fig. 6F,G). We found changes in ratios of splicing isoforms, indicating that SR proteins can directly or indirectly modulate EIS. Moreover, their effect on EIS is differential.

Next, we tested the regulation of EIS in the *SUPPRESSOR OF ABI* (*SUA*) mutant background. SUA, a homolog of the mammalian splicing factor RBM5 (Bonnal et al. 2008; Sugliani et al. 2010; O'Bryan et al. 2013), is responsible for the splicing inhibition of a cryptic intron in the *ABI3* gene (Sugliani et al. 2010). This cryptic intron is an internal part of a protein-coding exon; thus it qualifies as an exitron. We used two mutants, *sua-1* and *sua-2*, in the Ler-0 and Col-0 *A. thaliana* ecotypes, respectively (Sugliani et al. 2010). Consistent with a role of SUA in suppressing EIS in *ABI3*, the EI-spliced isoform ratio increased in the mutants (Fig. 3B; Supplemental Fig. 6H,I). Interestingly, EIS varies between Ler-0 and Col-0 ecotypes (Fig. 3B; Supplemental Fig. 6H,I) due to the presence of single-nucleotide polymorphisms (SNPs) affecting splicing signals (Fig. 3C). Considering the major effect of SUA on EIS, we checked whether exitrons contain binding motifs for RBM5 (Fushimi et al. 2008; Song et al. 2012). A genome-wide search for these motifs revealed their enrichment in exitrons (Fig. 3D). Conversely, a de novo motif search resulted in two motifs resembling the RBM5 motifs tested previously (Fig. 3D). Notably, these motifs show approximately fourfold enrichment in exitrons in comparison to IRs (Fig. 3D), implying differences in their splicing regulation. These findings suggest that SUA inhibits EIS in *Arabidopsis* by binding to exitrons, though the mechanism needs to be elucidated.

### Exitron-containing and exitron-spliced *Arabidopsis* transcripts are exported to the cytoplasm and are translated

It has been shown that IR transcripts are often retained in the nucleus (Göhring et al. 2014; Shalgi et al. 2014); however, there were also reports that IRs can be found in polysomal fractions (Ner-Gaon et al. 2004). Therefore, we addressed the fate of the transcripts resulting from EIS. As exitrons would have been classified previously as IRs, we examined published information on IR transcripts detected on ribosomes in *Arabidopsis* (Ner-Gaon et al. 2004). The only transcripts with retained introns within coding regions (AT2G18690, AT2G33340, AT3G13300, and AT4G01690) that were found to be associated with ribosomes (Ner-Gaon et al. 2004) are indeed transcripts with unspliced exitrons (Supplemental Table 1). Importantly, the EI-spliced isoforms of the AT3G13300 and AT4G07410 genes were also detected on ribosomes.

To validate translation of EI-containing and EI-spliced transcripts, we analyzed published *Arabidopsis* proteogenomic data sets generated from different organs, developmental stages, and cell cultures (Supplemental Results). We found peptides supporting both isoforms in three genes and EI-spliced isoforms in eight genes (Supplemental Table 3). In total, EI-spliced isoforms for 1.8% of all EI-containing genes are supported by peptides, and that compares well to the peptide support for 3.4% AS genes in mouse (Brosch et al. 2011). Our analyses show that both EI-containing and EI-spliced isoforms are exported to the cytoplasm and are translated, which contrasts the fate of other IR transcripts that are often retained in the nucleus (see Discussion).

### Exitron splicing affects protein function in *Arabidopsis*

**Figure 4.**
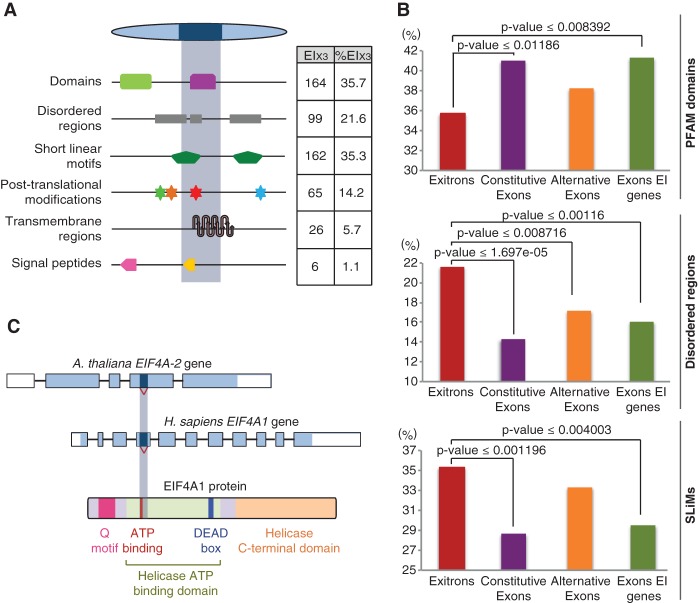
Functional implications of EIS in *Arabidopsis*. All analyses were performed for the EI_x3_ subset only. (*A*) Statistics of functional features of protein sequences encoded by EIs. (*B*) Enrichment of PFAM domains, disordered regions, and short linear motifs (SLiMs) in protein sequences encoded by EIs and different types of exons. (*C*) EIS removes the ATP-binding domain of the conserved eukaryotic translation initiation factor 4A in *Arabidopsis* and human.

As exitrons are protein-coding sequences, we next analyzed the consequences of EIS on protein features. The *Arabidopsis* EI_x3_s encode whole or parts of protein domains, disordered regions, post-translational modification (PTM) sites, transmembrane domains, and signal peptides (Fig. 4A; Supplemental Tables 9, 11–13), suggesting that EIS impacts the functional properties of the proteins. Indeed, our analysis of proteogenomic data supports EIS effect on the integrity of protein domains (Supplemental Tables 3, 9). Interestingly, for exitrons that overlap with protein domains, ∼35% of exitron boundaries coincide with domain borders (Supplemental Table 10). Though a considerable fraction (∼36%) of EI_x3_s affects protein domains (Fig. 4A), the latter are underrepresented in the EI_x3_ set when compared to constitutive exons or other exons of EI-containing genes (Fig. 4B). In contrast, we found an overrepresentation of disordered regions and short linear motifs (SLiMs) (Fig. 4B). A similar tendency was observed for human tissue-specific AS exons, suggesting their role in proteome versatility as disordered regions can influence protein conformation; carry SLiMs that bind to other proteins, DNA, RNA, and small molecules; and embed PTM sites (Buljan et al. 2013).

PTMs regulate protein functions by affecting their activity, localization, or affinity to other proteins. Our analysis of published experimental *Arabidopsis* PTM data sets shows that EI_x3_-encoded sequences carry sites for various PTMs (Supplemental Table 11). EIS can change sumoylation, ubiquitylation, S-nitrosylation, and lysine acetylation states of the protein isoforms, thus providing the first evidence that AS can influence protein function by affecting other types of PTMs besides phosphorylation (Zhang et al. 2010; Buljan et al. 2012; Merkin et al. 2012). Moreover, phosphopeptides are enriched in the EI_x3_-encoded sequences when compared to constitutive exons (11.3% vs 4.1%, *P*-value < 0.001), also when corrected by exitron and exon length (Supplemental Methods). Altogether, these results suggest that EIS impacts the dynamics of the *Arabidopsis* proteome.

The effect of EIS on protein features is illustrated by an EI_x3_ in the gene encoding the eukaryotic translation initiation factor 4A (EIF4A, AT1G54270), a DEAD-box RNA helicase (Fig. 4C). EIS removes the highly conserved ATP binding motif together with two conserved phosphorylation sites (Fig. 4C; Supplemental Fig. 7). Unwinding of substrates by this RNA helicase is ATP dependent (Cordin et al. 2006), implying that EIS affects this activity of EIF4A. This EIS event is supported by ESTs in *A. thaliana* and other plants (Supplemental Table 2; Supplemental Fig. 7). Moreover, an EST from fetal heart for the human *EIF4A1* shows EIS at the identical position as in *Arabidopsis* affecting the same highly conserved phosphorylation sites (Supplemental Fig. 7). This corroborates the importance of AS as a conserved strategy to modulate the phosphorylation status of proteins, as previously shown for mammalian alternative exons (Merkin et al. 2012). This high conservation suggests that EIS has an important regulatory function for EIF4A and was probably present before the divergence of plants and animals.

### Exitron splicing is an evolutionarily conserved strategy to increase versatility of transcriptomes

To find further cases of EIS events in plants, we produced a confident set of orthologous gene pairs using *A. thaliana* EI-containing genes (for numbers, see Fig. 7A) and tested it against respective EST collections. We found several conserved examples of EIS: in poplar POPTR_0002s23170, in grape Vv03s0038g03800, and in rice LOC_Os07g08729 and LOC_Os07g05570. The highest number of EIS events (46) was found in *Arabidopsis lyrata* (Supplemental Table 14). The low level of EIS detection can be explained by different depths of transcriptome coverage in these species (ESTs) in comparison to *A. thaliana* (RNA-seq), as observed for the discovery of AS events in general (Syed et al. 2012). To estimate EIS conservation, we used only *A. thaliana* and *A. lyrata* EST sets where the coverage is similar. Out of 98 genes with 100 EIS events supported by ESTs in *A. thaliana* (Supplemental Table 2), 56 genes have orthologs in our *A. lyrata* set. We found that 40 EIS events in these 56 genes (71.4%) are also supported by ESTs in *A. lyrata* (Supplemental Table 14). Deeper transcriptome data for plants will likely improve both EIS detection and the estimate of conservation levels. However, EIS can also be species-specific, thus providing a source for adaptation and speciation, as observed for AS exons (Barbosa-Morais et al. 2012; Merkin et al. 2012). In line with this, we found SNPs affecting EIS between two *A. thaliana* ecotypes (see above, Fig. 3B,C). Our further analysis of 82 ecotypes showed that 2.2% of SNPs either decrease or increase the strength of a splice site signal in at least one ecotype when compared to Col-0 (Supplemental Results; Supplemental Tables 5–8). As many EI-containing genes are involved in stress responses, the genetic variability affecting EIS could play a role in the adaptation of *A. thaliana* ecotypes.

EIS in the human *EIF4A1* indicates that these events are not restricted to plants. As exitrons could be classified as IRs, we searched for such cases in the literature. Indeed, we found IRs in the mammalian-specific genes human *CCKBR*, *CD55*, and *FMNL1* and mouse *Tgif2* that qualified as exitrons (Supplemental Table 15). Moreover, additional cases of splicing of intraexonic sequences were described in *Caenorhabditis elegans*, and a hypothesis on their origin was proposed (see below) (Irimia et al. 2008). Altogether, these findings demonstrate that EIS is a common strategy to increase transcriptome diversity in plant and nonplant species.

### Exitron splicing is a widespread alternative splicing event in human

**Figure 5.**
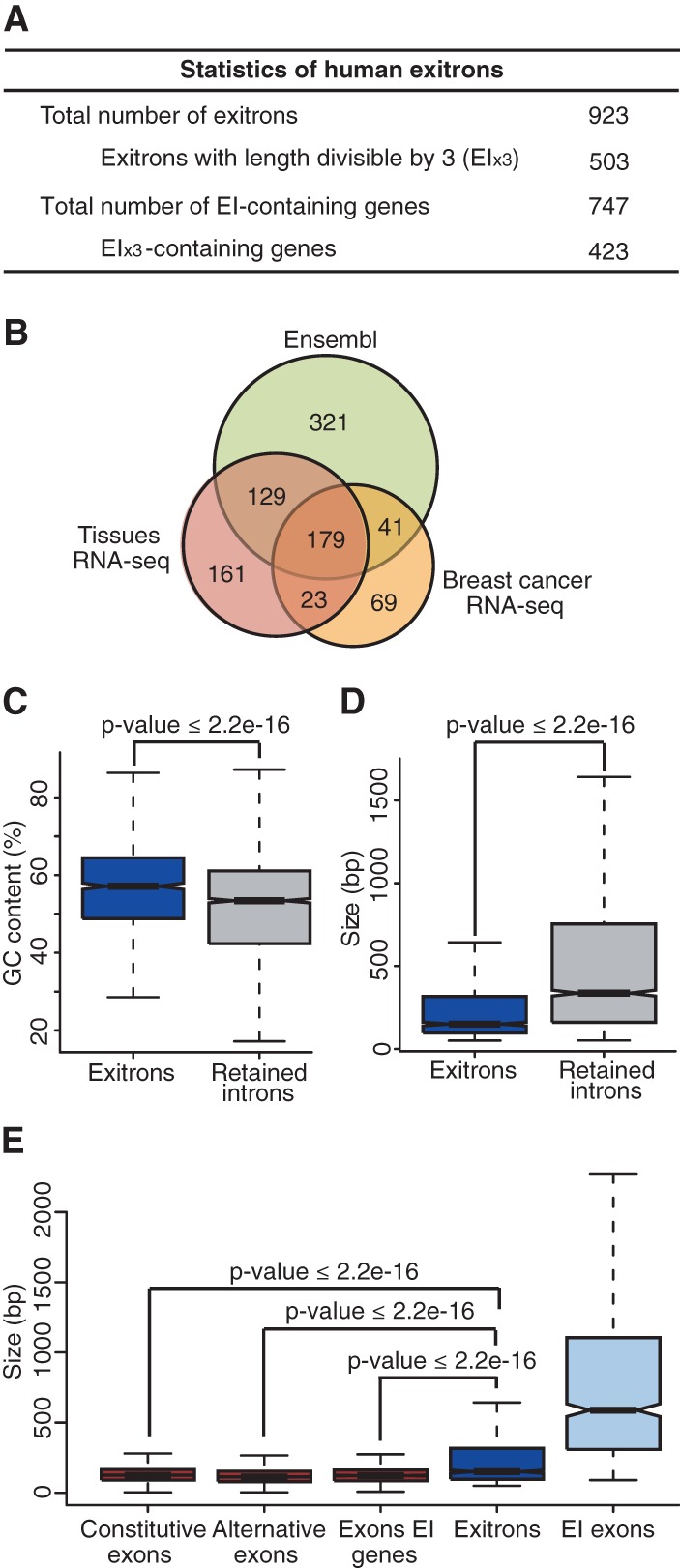
Identification and characterization of human EIs. (*A*) General statistics of human EIs. (*B*) Venn diagram of EIs identified by different sources. (*C*,*D*) Comparisons of GC content (*C*) and size distributions (*D*) of EIs and IRs. (*E*) Comparison of size distributions of EIs and different types of exons. (*C*–*E*) Data presented as Tukey box plots.

To obtain further evidence of EIS in nonplant species, we explored the set of annotated IR transcripts in human Ensembl. In this set, 670 retained introns (in 577 genes) qualified as exitrons (Supplemental Table 16), including the above-described exitrons in *FMNL1* and *CCKBR*, but not in *CD55*, suggesting that this set is not exhaustive. Thus, we analyzed RNA-seq data sets from six human tissues (brain, heart, liver, lung, ovary, and testis) (Barbosa-Morais et al. 2012) and from a ERBB2-positive breast cancer and the control breast tissue (NBS) (Eswaran et al. 2013). We found 602 exitrons in 488 genes (Supplemental Table 16). Altogether, we detected 923 EIS events in 747 genes (∼3.7% of 20,364 human protein-coding genes; GRCh37) (Fig. 5A), whereby 349 EIS events are shared between the Ensembl and RNA-seq exitron sets (Fig. 5B). Human EI_x3_-containing genes are enriched in genes with functions in DNA replication, immune response, and the mediator and calcium channel complexes (Supplemental Table 17). Similar to *Arabidopsis*, the 3n fractions of human exitrons (∼55%) and IRs (32.07%; based on analysis of RNA-seq and Ensembl data sets) are significantly different. Moreover, the 3n exitron fraction is much higher than the fraction of human 3n introns without stop codons (29.8%), previously shown to be counter-selected in the human genome (Jaillon et al. 2008). Human and *Arabidopsis* exitrons share other features, such as weaker splice sites and higher GC content in comparison to IRs; they have similar size distribution, and their sizes are closer to exons than to IRs (Fig. 5C–E; Supplemental Fig. 8A–C). In both species, EI-containing exons are considerably longer than other exons (Figs. 2D, 5E). Our analyses show that EIS affects a comparable number of genes in human and in *Arabidopsis*, and exitrons have similar features in both species.

### EIS affects protein properties in human

To obtain evidence for the translation of exitronic sequences in human, we analyzed published human tissue proteome data sets (Supplemental Methods). We found 382 peptides supporting 81 EI-encoded sequences (Supplemental Table 18), indicating that, as in *Arabidopsis*, EI-containing isoforms are exported to the cytoplasm and translated in contrast to IR transcripts that are often retained in the nucleus and not translated (Yap et al. 2012; Shalgi et al. 2014).

**Figure 6.**
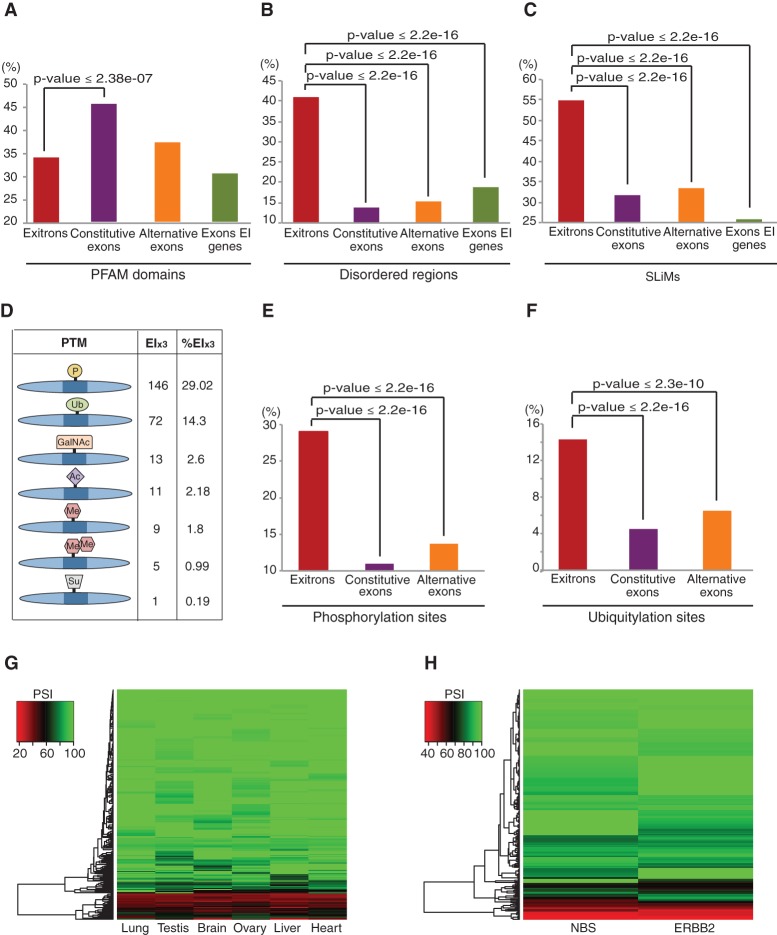
Functional implications of EIS in human. (*A*–*C*) Enrichment of PFAM domains (*A*), disordered regions (*B*), and SLiMs (*C*) in protein sequences encoded by EIs and other types of exons. (*D*) Statistics of various post-translational modification (PTM) sites encoded by EIs. (*E*,*F*) Enrichment of phosphorylation (*E*) and ubiquitylation (*F*) sites in the sequences encoded by EIs and other types of exons. (*A*–*F*) Analyses performed for EI_x3_ subset only. (*G*,*H*) Heatmap of EIS (measured by PSI) in different human tissues (*G*) and in ERBB2-positive breast cancer and normal breast tissue (NBS) samples (*H*).

Analyses of the human EI_x3_-encoded protein sequences showed that ∼34% overlap with protein domains, whereby splicing boundaries of about one-third of these EI_x3_s coincide with protein domain borders (Supplemental Tables 19, 20). Interestingly, EIS affects C2H2 type zinc finger (ZNF) domains in five KRAB-ZNF transcription factors that act as repressors of different endogenous retroviruses. Variation of their DNA binding specificity is achieved by gene duplication and recombination and by duplications and deletions of ZNF repeats that are organized in a single exon (Lukic et al. 2014). EIS affecting ZNF repeats can therefore provide another mechanism for a concerted evolution of combinatorial tools to inactivate retroviruses. As in *Arabidopsis*, protein domains are underrepresented, while disordered regions and SLiMs are enriched in human EI_x3_-encoded sequences (Fig. 6A–C). Similar to *Arabidopsis*, we also detected the first examples of AS affecting various PTM states of protein isoforms in human, whereby the list is expanded to methylation and O-linked glycosylation (Fig. 6D; Supplemental Table 21). Moreover, we detected enrichment not only for phosphorylation sites but also for ubiquitylation sites in the exitron-encoded sequences (see also Supplemental Methods; Fig. 6E,F; Supplemental Fig. 9). In addition, 710 PTM peptides provide further evidence for translation of 190 EI_x3_s in 161 genes (Supplemental Table 21). These findings indicate that EIS impacts protein features similarly in plants and humans, thus representing an evolutionarily conserved tool for shaping eukaryotic proteomes.

### EIS is differentially regulated across human tissues

We identified a total of 492 EIS events in six human tissue transcriptomes (Fig. 5B; Supplemental Table 16), whereby 217 (44.1%) are found in all samples. Analysis of the latter showed different PSI (percent of spliced in) values for EIS events across human tissues (Fig. 6G). EI-spliced isoforms can be predominant (PSI < 50) (Fig. 6G), however, as in *Arabidopsis*, EI-containing isoforms are the major transcripts in most cases as revealed by their high PSI values. As splice variants may only appear in a small number of cells types, the analysis of a whole human organ can underestimate the impact of an event. In addition, studies of more tissue samples and conditions would differentiate events with more ample regulation from those that may represent splicing noise.

We found 52 EIS events showing a change of ≥15% (ΔPSI ≥ 15) in at least one tissue, with 10 of them reported previously to have ample evidence for their regulation and physiological relevance (Supplemental Table 22). For example, EIS in the transcription factor *CIZ1* changes protein localization, and the EI-spliced isoform is up-regulated in Alzheimer's disease brains (Dahmcke et al. 2008). In agreement with these studies, we detected the EI-spliced isoform in all but the brain tissues (Supplemental Table 22). It is important to emphasize that previously reported EIS events comprise a wide range of ambiguous definitions such as intron retention, removing an intron from within the exon, intraexonic splicing, internal splicing event in exon, internal alternative splice sites, or cryptic 5′ and 3′ splice sites located in the exon (Supplemental Table 22), indicating uncertainty in the interpretation of the type of AS event.

For EIS events with a PSI ≤ 90 in at least one tissue, we observed their differential distribution across human tissues (Supplemental Table 23). The fraction of EIS events in testis (59.4%) is twice as high as in lung (29.2%), heart (27.7%), or liver (26.6%), while being intermediate in ovary and brain (43.5% and 42.4%, respectively). This distribution differs considerably from frequencies of other types of AS events. Usage of A5SS and A3SS is the most prominent in liver, and ES events are the most frequent in brain and testis; this was attributed to tissue-specific combinations and levels of splicing factors regulating particular events (Yeo et al. 2004). It has been suggested that the high number of AS events in testis may be due to splicing noise as many of these events are not conserved between mouse and human (Kan et al. 2005). However, these events can be species-specific, and AS could be one of the mechanisms driving rapid evolution of the reproductive systems (see also Discussion) (Elliott and Grellscheid 2006). A number of EIS events were detected only in a given tissue (25 in brain, 11 in heart, seven in liver, 13 in lung, 17 in ovary, and 44 in testis) (Supplemental Table 23). However, analyses of more samples are required to determine whether they are indeed tissue-specific. Altogether, our results indicate that EIS is tissue regulated and contributes to shaping the human tissue transcriptomes.

### Exitron splicing is misregulated in breast cancer

AS is linked to numerous human diseases, including cancer, suggesting its critical role in organism homeostasis (Srebrow and Kornblihtt 2006; Kelemen et al. 2013). By inspecting the human exitron list, we found that EIS affects several cancer-related genes: the cancer marker genes *BMI1*, *KRT5,* and *MUC1* and the genes involved in cell adhesion (*CSF1*), migration and metastasis (*ZEB2* and *KLF17*) (Supplemental Table 16).

To address a role for EIS in carcinogenesis, we analyzed ERBB2-positive breast cancer and normal breast tissues (NBS). Out of a total of 312 EIS events (Supplemental Table 16), 275 are detected in both samples. The PSI values for the latter differ between ERBB2 and NBS (Fig. 6H), with 29 having a ΔPSI ≥15 (Supplemental Table 24). These include EIS in the *FOSB* gene, resulting in the delta *FOSB* isoform, consistent with previous reports on its differential AS in breast carcinomas (Milde-Langosch et al. 2003). Though *FOSB* is well characterized, the type of AS event was defined ambiguously, including terms like “intronic” sequence in ORF of exon 4 or intron retention (Supplemental Table 24). This intron classifies as an exitron: It is protein-coding and without stop codons, and since its length is not a multiple of three, the stop codon is introduced only upon its splicing. AS in *FOSB* is regulated by the splicing factor PTB1 competing with U2AF65 for binding to the 3′ end of the exitron described as retained intron 4 (Marinescu et al. 2007). Interestingly, EIS in *Arabidopsis* is suppressed by SUA that interacts with U2AF65, potentially interfering with early spliceosome formation (Sugliani et al. 2010). Similarly, PTB1 can inhibit EIS events by competing with U2AF65 and preventing spliceosome assembly.

Remarkably, many EI-containing genes with differential EIS in the ERBB2 sample were shown to play a role in cancer (Supplemental Table 24), implying that an impairment of EIS can contribute to carcinogenesis. In 12 of these cases, EIS affects phosphorylation, ubiquitination, methylation, or acetylation of the resultant protein isoforms, suggesting a cross-talk of AS and PTM in breast cancer. Additional functional characterization of the EIS events in different cancer types may contribute to finding novel biomarkers for cancer diagnosis or therapy.

### A subset of exitrons evolved from ancestral exonic coding sequences

**Figure 7.**
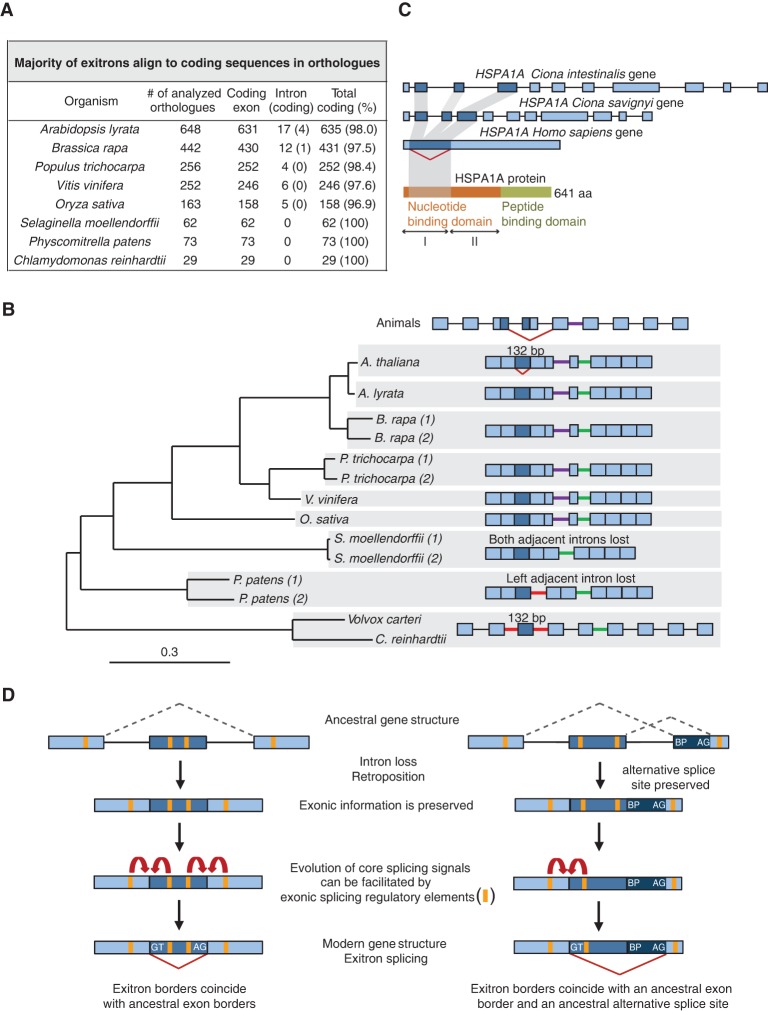
Origin and evolution of EIs. (*A*) Statistics of EI alignments to plant orthologous sequences. (*B*) EI evolution in the gene encoding glycine cleavage system T-protein. Phylogenetic reconstruction of intron loss events in different plant species (1 and 2 indicate paralogs). Gene structures are not to scale. Introns at the conserved positions are colored. EI and the homologous sequences are in dark blue. Red carets, EIS and ES events. (*C*) EI evolution in the intronless human *HSPA1A* gene. The human EI corresponds to three coding exons in *Ciona* spp. (*D*) Evolution of EIS, the “splicing memory” hypothesis. Evolution of a subset of EIs involved loss of introns and retroposition. Upon intron loss, exonic information was preserved. If ancestral regions were subjected to alternative splicing (dashed lines), vestigial exonic splicing regulatory elements present close to the former exon borders could facilitate evolution of core splicing signals and the reestablishment of an AS event in the modern gene by EIS.

That exitrons are not canonical introns raises the question of their evolutionary origin. We tested two of the potential scenarios. In the first scenario, exitrons could originate from ancestral introns. If so, then exitrons would be classical, noncoding introns in orthologous genes. Alternatively, exitrons could stem from ancestral protein-coding regions. In this case, the exitrons would correspond to protein-coding regions in orthologs. We did not find any cases of distant orthologs (*Chlamydomonas reinhardtii*, *Physcomitrella patens*, and *Selaginella moellendorffii*) in which exitrons corresponded to introns. Almost all *A. thaliana* exitrons align to exonic protein-coding sequences in the sets of analyzed orthologs (Fig. 7A; Supplemental Table 26). Up to 3% of exitrons align to introns that may have lost their coding capacity in the modern species. Alternatively, they may represent evidence for ancestral introns that acquired a coding capacity. In addition, other possible scenarios of exitron origin can exist that are not tested by this approach and can be explored in future. As exitrons are internal regions of protein-coding exons, selective pressure would prevent substitutions that disrupt their coding capacity, while favoring those that generate splicing signals and facilitate splicing. Indeed, SNPs in the exitron regions in 82 *Arabidopsis* ecotypes (Supplemental Results) are more frequent at the third (synonymous) positions of codons, as expected for coding sequences. Moreover, analyses of the 252 exitrons that reside in genes with paralogous copies showed the same tendency of substitutions (Supplemental Fig. 10). This contrasts the pattern usually found in introns, with no such preference for substitutions. Importantly, the analysis of paralogs revealed that substitutions in exitrons lower their GC content and increase splice site scores (Supplemental Table 27). These results show that the majority of exitrons in the analyzed set of orthologs and paralogs originate from ancestral protein-coding sequences that acquired the capacity of being spliced out.

### The evolution of a set of exitrons involved loss of introns

Several observations suggested that intron loss could have played a role in EIS evolution. First, *Arabidopsis* and human exitron boundaries often coincide with protein domain borders (Supplemental Tables 10, 20), reminiscent of the strong correlation observed between the borders of exons and protein domains (Liu and Grigoriev 2004). Second, EI-containing exons are longer than other exons (Figs. 2D, 5E). Third, many *Arabidopsis* and human exitrons reside in annotated intronless genes (Supplemental Tables 1, 25), including 29 *Arabidopsis PPR* genes, which have evidence of retroposition and intron loss in flowering plants (O'Toole et al. 2008). Therefore, we examined the regions corresponding to *A. thaliana* exitrons in paralogs and orthologs for the presence of introns. We found evidence of introns in paralogs for 22 exitrons (out of 252) (Supplemental Table 28). Analysis of orthologous gene structures in eight different plant genomes (for numbers of analyzed orthologs, see Fig. 7A) shows that 109 regions corresponding to 54 *A. thaliana* exitrons are interrupted and/or bordered by introns in the orthologs (Supplemental Table 29). The highest evidence of intron presence was detected in the most distant species: 65.5%, 24.7%, and 11.3% cases of analyzed orthologs in *C. reinhardtii*, *P. patens*, and *S. moellendorffii*, respectively. In contrast, *A. lyrata* showed the lowest number of introns in the regions corresponding to exitrons, probably because of a high number of conserved exitrons (based on analysis of ESTs; see above). Further analysis of intron positions revealed interesting features. First, for 16 exitrons, we found 28 orthologous gene structures that support the presence of introns exactly or close (≤10 nt) to one or both exitron borders in *A. thaliana* (Supplemental Table 29). Second, an exitron can correspond not only to a single exon but also to multiple exons in other species. For example, the exitron in *Arabidopsis THIC* gene corresponds exactly to three exons in *C. reinhardtii* (Supplemental Fig. 11A). The exitron in the gene encoding the CBS domain-containing protein comprises four and one-half exons in paralogs and orthologs, whereby intron positions in these genes are highly conserved. Interestingly, we found that the region corresponding to this exitron undergoes AS in the paralog (Supplemental Figs. 11B,C).

EIS evolution in plants is illustrated by a gene coding for the highly conserved T-protein, a component of the glycine cleavage system. Interestingly, EIS in *Arabidopsis* overlaps with a conserved ES event in the orthologous *AMT* gene in animals (Fig. 7B; Supplemental Fig. 12A,D). Both AS events remove the region involved in the enzymatic activity, thus resulting in a very similar functional outcome. Structures of plant orthologs show that the exitron corresponds to an exon of the same size precisely bordered by canonical introns in two green algae species (Fig. 7B; Supplemental Fig. 12A,B). Further analysis revealed synonymous substitutions favoring the appearance of exitron splice sites in the *Arabidopsis* genus (Supplemental Fig. 12C). Interestingly, highly conserved short sequences with avoidance of substitutions are present close to the exitron (Supplemental Fig. 12C) that might represent splicing regulatory elements (Fairbrother et al. 2004). Their analysis shows that they are potential binding sites for RBM5/SUA and CELF2 (Supplemental Fig. 12C). Interestingly, the binding specificity of CELF2 homologs is conserved from human to *Arabidopsis*, and these proteins have multiple functions in RNA processing, including AS (Good et al. 2000; Kim et al. 2013).

In human, intron loss during EIS evolution is illustrated by the annotated intronless gene *HSPA1A* (*HSP70A-1*) (Fig. 7C; Supplemental Fig. 13). The EIS event removes the first of two subdomains of the ATPase domain of HSPA1A. This exitron corresponds exactly to three exons in the *Ciona* sea squirt species. Interestingly, GT and AG dinucleotides are already present at the corresponding exon borders in *Ciona*.

Altogether, our findings suggest that intron loss accompanied the evolution of a subset of EIS events. A full in-depth study of *Arabidopsis* and human EI-containing genes should be performed in the future to estimate the impact of intron loss on exitron splicing evolution.

## Discussion

We have performed a comprehensive characterization of a subfamily of AS events, the splicing of exitrons, that allows intraexonic protein-coding sequences to be differentially spliced. Previously, we named them cryptic introns (Marquez et al. 2012); however, their hybrid nature combining features of introns and protein-coding exons is reflected better in the new term, exitrons (exonic introns). It will avoid any confusion with cryptic splice sites that are activated by mutations disrupting the usage of the natural splice sites (Roca et al. 2003). Moreover, while some of EIS events were detected previously, their description in published literature has been ambiguous (Supplemental Tables 15, 22, 24; Ner-Gaon et al. 2004). These events were defined as intron retention, as an internal splicing event in the exon, or as usage of cryptic splice sites located in the exon, all reflecting uncertainty in the interpretation of the type of AS event.

The categorization of AS events has promoted studies of mechanistic differences in splicing regulation and their contribution to phenotype. Recent classification of IRs based on their evolutionary origin and conservation has defined a minor group (type B) of IRs located within exons, including noncoding ones (Braunschweig et al. 2014). Though very useful in terms of evolution, this approach fails to detect species-specific AS events. The latter diverge strongly even between closely related species (∼50% of human and chimp AS exons are different), thus contributing to phenotypic differences (Barbosa-Morais et al. 2012; Merkin et al. 2012). As the definition of exitrons is not based on their evolutionary origin, it overcomes this issue and allows detecting this AS event without the need to compare transcriptomes of different species. The only requirement to define exitrons is the protein-coding potential of EI-containing (unspliced) isoform. While our definition does not include events in noncoding regions, it provides a more homogeneous set of AS events for evolutionary, functional, and mechanistic studies.

Though exitrons were found in the IR sets of *Arabidopsis* (Marquez et al. 2012) and human (Ensembl IR-annotated transcripts), their separation is important, as they have clearly distinguishable features and, notably, their splicing results in transcripts with different fates. First, EI-containing transcripts associate with ribosomes and are translated. Splicing of EIs affects essential protein features. In contrast, intron retention is suggested to be a mechanism to forestall translation, when IR transcripts are recognized as incompletely processed and remain in the nucleus until removal of retained introns post-transcriptionally (Boothby et al. 2013; Shalgi et al. 2014). Second, a PTC can be created downstream from an exitron (non EI_x3_) only upon splicing, while in the case of IRs, PTCs are generated due to splicing inhibition and retention of intronic sequences. Moreover, while such EIS events can result in NMD-sensitive transcripts, IR transcripts, though possessing PTCs in a NMD-sensible context, avoid the NMD machinery, at least in *Arabidopsis* (Kalyna et al. 2012; Leviatan et al. 2013), probably due to their retention in the nucleus (Göhring et al. 2014). Third, EI-containing transcripts are major isoforms as evidenced by high PSI values, while IR isoforms are usually of low abundance (Marquez et al. 2012; Braunschweig et al. 2014). And fourth, we demonstrate that a subset of exitrons originates from protein-coding exons. Therefore, it is not surprising that they display features characteristic for such sequences (high GC content, absence of stop codons, overrepresentation of EI_x3_s, and the prevalence of synonymous substitutions); these features are totally atypical for IRs or any type of intron. All this clearly separates exitrons from IRs, while the impact of EIS on the proteome is more similar to skipping of protein-coding exons.

It is well documented that chromatin state, nucleosome positioning, RNA polymerase II occupancy and processivity, and binding of splicing factors differ between exons and introns, impacting AS regulation (Braunschweig et al. 2013; Reddy et al. 2013). Therefore, the unique features of exitrons as intraexonic sequences imply distinct mechanisms controlling their splicing; consequently, a clear differentiation of exitrons from IRs is relevant for studies on AS regulation. In addition, finding EIS in annotated intronless genes revises the concept that such genes are devoid of splicing regulatory elements, further impacting AS research.

Our finding of EIS raises the question of how internal, essentially exonic, regions have evolved into exitrons. It has been proposed that mutations in protein-coding sequences, creating a PTC, would promote intronization of the affected region to rescue at least a shortened ORF (Catania and Lynch 2008). EIS evolution must have proceeded differently because exitrons do not contain PTCs. On the contrary, splicing of non-EI_x3_ may actually result in PTCs, albeit downstream from EIS events (Fig. 1). Cases of intronization of exonic sequences in different species have been described previously (Irimia et al. 2008; Zhu et al. 2009; Kang et al. 2012; Braunschweig et al. 2014). It has been suggested that intronization can occur due to single substitutions creating GT/C and AG splicing boundaries (Irimia et al. 2008). In this hypothesis, it is not clear what the driving force for intronization is. Additionally, though these dinucleotides are required at splice sites, they do not create the complete splice site signals. Furthermore, these substitutions are not always needed, as they can be already present in the ancestral sequences (as in *Ciona HSPA1A* orthologs). Moreover, numerous cryptic splice sites can be present in the pre-mRNA, but they are rarely if ever used (Wang and Burge 2008). Interestingly, in human retrogenes, such cryptic splice sites can be activated due to loss of oppression upon retroposition, thus leading to intronization of exonic sequences (Kang et al. 2012). As such promiscuous splicing is not functionally relevant, it can be detrimental, explaining why these new introns are mainly observed in pseudogenes. This differs from exitrons because they are under positive selection to preserve their coding potential. However, contribution of such cryptic splice sites to the evolution of some exitrons cannot be excluded and needs to be further investigated. Our findings led us to the idea that evolution of at least some exitrons could be a consequence of intron loss, especially at the exitron borders. The potential to restore splicing after intron loss is corroborated by recursive splicing of the *Drosophila Ubx* gene, where the splice sites are regenerated at the exon–exon junctions after intron splicing (Hatton et al. 1998). However, it is obvious that exitron splicing does not occur in every region or gene that lost introns. Therefore, we propose a “splicing memory” hypothesis to explain the evolution of exitron splicing. Genes, upon intron loss and retroposition, have footprints of former exon borders and thus “remember” previously defined exons (Fig. 7D). Exonic splicing regulatory sequences at the proximity to the exon borders required for splice site selection (Reed and Maniatis 1986) can constitute such footprints and can contribute to EIS evolution. If ancestral exons were alternatively spliced, then vestigial exonic splicing regulatory elements could provide the position-dependent information on ancestral AS patterns (“splicing memory”). We found highly conserved short sequences close to the exitron in the T-protein genes that can potentially represent binding sites for RNA processing factors with functions beyond splicing. Binding of such proteins to the motifs still present in the exonic sequences may connect a region that no longer contains introns to the RNA processing and splicing network. Tethering the spliceosomal components to these regions could favor mutations beneficial for retrieval of the splicing-relevant information in response to some cue and support the emergence of splicing signals, thus restoring production of AS transcripts via EIS. This hypothesis would also apply to intronization events in noncoding regions. Interestingly, the highest number of EIS events was detected in the testis. The heritable intron loss mediated by retroposition is limited to germline cells or their embryonic precursor cells and to the genes expressed in these cells (Roy and Gilbert 2006). Therefore, our hypothesis for the evolutionary origin of exitrons that involves intron loss can potentially explain the high number of EIS events detected in the testis.

Nevertheless, in-depth studies of intron loss, ancestral AS events, conserved splicing regulatory elements, and a potential role of RNA processing factors should be performed in the future to test this hypothesis. In addition, since our hypothesis is based on analyses of a limited set of EI-containing genes, other scenarios for exitron splicing evolution cannot be excluded.

## Methods

### Exitron sets, features of exitrons, and exitron-containing genes

The set of *A. thaliana* exitrons was obtained using our RNA-seq of normalized cDNA library prepared from flowers and 10-d-old seedlings (Marquez et al. 2012). Human exitrons were identified using Ensembl-annotated intron retention transcripts (GRCh37; Flicek et al. 2013) and RNA-seq data sets for the brain, heart, liver, lung, ovary, and testis (Barbosa-Morais et al. 2012) and for breast organoids (epithelium) samples (NBS) and type ERBB2-positive breast tumor (Eswaran et al. 2013). The new release of the human genome annotation (GRCh38) does not affect the conclusions of our study as (1) the human exitron set has been obtained using CCDS (Consensus CDS) annotation (see Supplemental Methods), and (2) analyses of two very different genomes (*A. thaliana* and *H. sapiens*) show that *Arabidopsis* and human exitrons have very similar features and their splicing results in similar functional consequences at the protein level (see Results). *Arabidopsis* and human retained introns, constitutive and alternative exons are derived from our RNA-seq data (Marquez et al. 2012) and Ensembl, respectively. Mann-Whitney-Wilcoxon tests were used for test differences in GC content and size distributions. For details, see Supplemental Methods. GO classification for *Arabidopsis* and human EI_x3_-containing genes was performed with the classification SuperViewer (http://bar.utoronto.ca/ntools/cgi-bin/ntools_classification_superviewer.cgi) and GOEAST (http://omicslab.genetics.ac.cn/GOEAST/tools.php; Zheng and Wang 2008) tools, respectively.

### Validation of *Arabidopsis* exitron-spliced isoforms

*Arabidopsis* exitron-spliced isoforms were validated by in vitro transcription, EST data sets (PlantGDB [Duvick et al. 2008] and The Arabidopsis Information Resource [TAIR]), the high-resolution RT-PCR panel, Sanger sequencing, and conventional RT-PCRs (see Supplemental Methods).

### Validation of translation of EIS transcripts

Translation of EI-containing and EI-spliced isoforms was verified in *Arabidopsis* with two proteogenomic data sets (Baerenfaller et al. 2008; Castellana et al. 2008), and for human, www.proteomicsdb.org (Wilhelm et al. 2014) was used (see Supplemental Methods).

### Analyses of exitron splicing regulation

For *Arabidopsis*, RT-PCRs were done using RNA extracted from different tissues, stress conditions, and genetic backgrounds. In human, PSI values were calculated to determine differential EIS. RBM5 experimental binding motifs were obtained from Fushimi et al. (2008) and Song et al. (2012). De novo motif discovery in the exitron sequences was performed with MEME from the MEME suite (http://meme.nbcr.net/meme/; Bailey et al. 2009). Binomial tests were applied for testing motif enrichment. See also Supplemental Methods.

### Analysis of the impact of the exitron splicing at the protein level

PFAM protein domains, disordered regions and SLiMs were predicted using HMMER (Finn et al. 2011) (PFAM 25.0), IUPRED (short mode) (Dosztanyi et al. 2005), VSL2B (Obradovic et al. 2005; Peng et al. 2006), and the ANCHOR program (Dosztanyi et al. 2009), respectively. PTM experimental sets for *A. thaliana* are reported in Supplemental Table 11. Binomial tests were applied for enrichment of the protein features between exitrons and the different groups of exons. See also Supplemental Methods.

### Genetic variation in exitron sequences in *A. thaliana* ecotypes

SNPs of 82 natural *A. thaliana* ecotypes were mapped against exitron sequences (see Supplemental Methods). The impact of SNPs in the 5′ and 3′ splice site signals was evaluated using PWMs. The position of the SNP in the codon was determined using the phase of the EI-containing exon.

### Evolutionary studies of exitrons

Paralogs and orthologs of *Arabidopsis* EI-containing genes were obtained from Bolle et al. (2013) (http://www.gabi-kat.de/db/duplo_genepairs.php) and by using a bidirectional best-hit approach, respectively. Plant EST data sets were obtained from The Joint Genome Institute (http://genome.jgi-psf.org/Araly1) and from PlantGDB (http://www.plantgdb.org). For human exitron evolution, the presence and positions of introns in the orthologs were analyzed using Ensembl Compara resources and tools. See also Supplemental Methods.
